# Association of Body Composition with Outcome of Docetaxel Chemotherapy in Metastatic Prostate Cancer: A Retrospective Review

**DOI:** 10.1371/journal.pone.0122047

**Published:** 2015-03-30

**Authors:** Weixin Wu, Xiandong Liu, Patrick Chaftari, Maria Teresa Cruz Carreras, Carmen Gonzalez, Jayne Viets-Upchurch, Kelly Merriman, Shi-Ming Tu, Shalini Dalal, Sai-Ching J. Yeung

**Affiliations:** 1 Department of Emergency Medicine, The University of Texas MD Anderson Cancer Center, Houston, Texas, United States of America; 2 Department of Genitourinary Medical Oncology, The University of Texas MD Anderson Cancer Center, Houston, Texas, United States of America; 3 Department of Symptoms Control and Supportive Care, The University of Texas MD Anderson Cancer Center, Houston, Texas, United States of America; 4 Department of Endocrine Neoplasia and Hormonal Disorders, The University of Texas MD Anderson Cancer Center, Houston, Texas, United States of America; University of Cordoba, SPAIN

## Abstract

**Background:**

Docetaxel, a lipophilic drug, is indicated for castration-resistant metastatic prostate cancer. Most men with such disease would have had androgen-deprivation therapy, which decreases muscle and increases body fat. Obesity and body composition changes may influence the outcomes of docetaxel therapy.

**Methods:**

We conducted a retrospective review of 333 patients with metastatic prostate cancer treated with docetaxel at a comprehensive cancer center between October 7, 2004 and December 31, 2012. Body composition parameters were measured based on the areas of muscle and adipose tissues in the visceral and subcutaneous compartments on CT images at L3-4 levels. Dose calculations, toxicity and adverse reaction profiles, and overall survival were analyzed.

**Results:**

Obese patients were younger at the diagnosis of prostate cancer and had a shorter duration from diagnosis to docetaxel therapy. Analysis of body composition found that a high visceral fat-to-subcutaneous fat area ratio (VSR) was associated with poor prognosis but a high visceral fat-to-muscle area ratio (VMR) and high body mass index were associated with increased duration from starting docetaxel to death, allowing such men to catch up with patients with normal body mass index in overall survival from cancer diagnosis to death. Cox proportional hazard regression showed that age ≥65 years, high VSR, abnormal serum alkaline phosphatase, and >10% reduction of initial dosage were significant predictors of shorter time between starting docetaxel and death, and that high VMR, obesity, and weekly regimens were significant predictors of longer survival after docetaxel.

**Conclusion:**

Obese and overweight patients may benefit more from weekly docetaxel regimens using the reference dosage of 35 mg/m^2^ without empirical dosage reduction.

## Introduction

Prostate cancer is the most commonly diagnosed cancer in men in the United States and the second most common worldwide. In men with metastatic or recurrent prostate cancer, androgen-deprivation therapy (ADT) is first-line therapy to reduce morbidity and improve survival [1]. The hypogonadal state changes body mass composition. ADT given for 12 months significantly decreases muscle and bone mass and increases fat mass, resulting in a net weight gain [2, 3]. A longitudinal study has shown that prostate cancer patients on ADT gain about 2.2 kg in weight during the first year of therapy and then remain stable at that higher weight thereafter [4]. In addition, aging and a decline in physical activity also contribute to changes in body composition.

The management of castration-resistant metastatic prostate cancer after ADT remains a major clinical challenge, because patients often have pain and progressive decline in performance status. Currently, advanced or symptomatic castration-resistant metastatic prostate cancer is often treated with docetaxel [5, 6]. The US FDA-approved docetaxel dose for castration-resistant metastatic prostate cancer is 75 mg/m^2^ given intravenously over 1 hour every 21 days on Day 1 for 10 cycles [7]. Alternatively, docetaxel can be given at 50 mg/m^2^ every 2 weeks [8]. Weekly docetaxel dosing is given at 35 mg/m^2^, [9] 36 mg/m^2^, [10] or 40 mg/m^2^ [11] intravenously weekly for 6 weeks followed by a 2-week recovery period. Comparison of docetaxel pharmacokinetics in the weekly and triweekly regimens showed that they are similar [12].

Doses of chemotherapy are usually based on the body surface area (BSA), which considers weight and height. However, dosing based on BSA is not very useful in reducing inter-patient variability in drug clearance [13]. Various drug elimination processes, e.g., metabolic breakdown or excretion, account for inter-patient variability in pharmacokinetics to a large degree [14]. Body composition (adipose tissue and muscle mass) [15] is another factor influencing pharmacokinetics and may predict toxic reactions to certain chemotherapy regimens [16, 17]. The absolute clearance of docetaxel is not significantly changed by obesity as classified by body mass index (BMI), and empirical strategies for dose adjustments in obese patients are not warranted [15]. However, the influence of detailed body composition parameters on docetaxel pharmacokinetics has not been fully investigated. In a retrospective study of breast cancer patients, obesity was associated with a reduction in docetaxel dose intensity [18]; however, the association of body composition with reduction in docetaxel dose intensity in prostate cancer patients has not been explored. Although the American Society of Clinical Oncology (ASCO) has recommended that chemotherapy doses for obese patients should not be reduced because of the risk of compromising treatment efficacy and the lack of evidence for increased toxicity [19], the studies that contributed to these recommendations did not involve docetaxel.

We hypothesized that the body composition of patients with castration-resistant metastatic prostate cancer may influence clinical outcomes and toxicity of docetaxel treatment. Therefore, we performed a retrospective review of metastatic prostate cancer treated with single-agent docetaxel therapy in patients with CT scans of the abdomen available for analysis of body composition. The association of body composition parameters with differences in clinical outcomes and toxicity in castration-resistant metastatic prostate cancer patients was examined.

## Materials and Methods

### Study Population

This retrospective study was approved by the Institutional Review Board of The University of Texas MD Anderson Cancer Center Institutional Review Board in accordance with an assurance filed with, and approved by the Department of Health and Human Services. No informed consent was required for this retrospective review, and all patient records/ information were anonymized and de-identified prior to analysis. Using the Tumor Registry, diagnostic imaging records, and the pharmacy dispensing records of MD Anderson Cancer Center, we identified 378 consecutive prostate cancer patients who were treated with docetaxel and who had a CT scan of the abdomen and pelvis within 30 days of initiation of docetaxel between October 7, 2004 (the date of publication by Tannock et al. [5]) and December 31, 2012. The following exclusion criteria were applied to these patients: i) concurrent treatment with other cytotoxic chemotherapeutic agents (e.g., estramustine) or targeted therapy (e.g., imatinib, or sunitinib), ii) lack of confirmation of metastatic disease, iii) incomplete medical records, iv) histological types of prostate cancer other than adenocarcinoma (e.g., small cell cancer), and v) incompatible digital image formats of CT scans. The final study cohort consisted of 333 patients.

### Clinical Data Collection

Trained research personnel reviewed records to collect information on demographics and known or suspected risk factors for prostate cancer prognosis (i.e., age, Gleason grade, black race). Because black race is an adverse prognostic factor for prostate cancer [20], the race of this patient cohort was categorized as black vs. non-black. The pathologic diagnosis and Gleason grade (the sum of the scores) of the primary tumor were recorded. Because all patients in this cohort had metastatic disease at the time of initiation of docetaxel chemotherapy, the TNM stage of the prostate cancer is not relevant to this study. Clinical data for each patient were reviewed to assess the age-non-adjusted Charlson Comorbidity Index (CCI) [21].

### Chemotherapy

All patients received single-agent cytotoxic chemotherapy with docetaxel. The chemotherapy was administered intravenously every 3 weeks, as approved by the FDA, or weekly with some variations in the scheduling of the break week(s). Docetaxel doses were calculated based on BSA[22] and might be modified or reduced at the discretion of the treating oncologist. The initial BSA-based dosages were compared with reference doses of 35 mg/m^2^ for weekly regimens [9] and 75 mg/m^2^ for the non-weekly regimens [7]. In cases of excessive toxicity, treatment adaptations consisted of dose reductions or chemotherapy disruption or discontinuation.

### Toxicity evaluation

The tolerance to chemotherapy was evaluated before each cycle by clinical examination and a complete blood count. We ascertained (by review of clinical records and laboratory results) the presence of significant neutropenia (defined as absolute neutrophil count <1000/mm^3^), complaints of diarrhea, nausea and vomiting, peripheral neuropathy, mucositis, allergic reaction/anaphylaxis, and hand and foot syndrome. A side effect or adverse reaction was considered present if it was explicitly mentioned or discussed in the medical records; otherwise, it was considered absent or not important. Because of the risk of bias in the evaluation of non-hematological toxicities assessed retrospectively, the analysis of these toxicities was considered exploratory.

### Dose intensity evaluation

All events related to docetaxel administration were reviewed, i.e., dose reductions, delayed chemotherapy, changes in dosing regimen, or discontinuation of chemotherapy. When there was a reduction in dosage intensity, the new relative dose was calculated by the new dose intensity (mg/m^2^/week) divided by the original dose intensity (mg/m^2^/week) of docetaxel.

### Follow-up

The primary clinical outcome was overall survival calculated from the start of docetaxel therapy. Survival information was obtained through the Tumor Registry of MD Anderson Cancer Center. Methods of follow-up for the Tumor Registry include letters and phone calls, computer matches with the business office for kept appointments, searches of public databases (the Social Security Death Index, Bureau of Vital Statistics of Texas and neighboring states), and MD Anderson clinic staff notifications. If a patient was not known to be dead, survival time was censored at the last follow-up. The overall survival duration was defined as the duration between initiation of docetaxel therapy and death or last contact.

### Body composition analysis

BMI (kg/m^2^) was calculated using the recorded height and body weight closest to the date of the CT scan and categorized according to the World Health Organization standard: BMIs of 18.5–24.9, 25–29.9, and ≥30 kg/m^2^ were defined as normal body weight, overweight, and obesity, respectively. To investigate the relationship of body composition of patients with metastatic prostate cancer and docetaxel chemotherapy, CT scans of the abdomen and pelvis within 1 month from the initiation of docetaxel were analyzed. NIH ImageJ (version 1.47, http://imagej.nih.gov/ij/) was used to analyze cross-sectional images at L3-4 levels as described by Richards et al. [23] Two different images underwent fat contouring and were measured by a reviewer; two different additional images were measured by a different reviewer; results from the four images were then averaged. Total fat, subcutaneous fat, visceral fat, and skeletal muscle cross-sectional areas (cm^2^) were measured. Each parameter was then normalized for the square of the body height as in BMI and designated as iTAT, iSAT, iVAT, and iSKM, respectively, each with the unit cm^2^/m^2^. The visceral fat-to-muscle ratio (VMR) was obtained by dividing visceral fat area by skeletal muscle area; the visceral fat-to-subcutaneous fat ratio (VSR) was obtained by dividing visceral fat area by subcutaneous fat area. Patients were considered sarcopenic if they had a lumbar iSKM of ≤53 cm^2^/m^2^ for men with BMI ≥25 kg/m^2^ and of ≤43 cm^2^/m^2^ for men with BMI<25 kg/m^2^ as previously described [24]. The volume of skeletal muscle (SKM) and total adipose tissue (TAT) (in liters) was estimated using the following formulae: SKM = 0.068 × total adipose tissue cross-sectional area in cm^2^ at L3-4 level + 2.142; and TAT = 0.166 × skeletal muscle cross-sectional area in cm^2^ at L3-4 level + 4.142, respectively [25]. The volumes of subcutaneous adipose tissue (SAT) and visceral adipose tissue (VAT) were estimated based on the VSR. The doses of docetaxel per liter of estimated SKM, TAT, SAT, and VAT were calculated.

### Statistical Analysis

Baseline patient characteristics, clinical risk factors, and prostate cancer prognosis were compared between groups by using the chi-square test, Fisher’s exact test, Student’s t-test, or Mann-Whitney rank sum test where appropriate. For comparisons among multiple groups, parametric or nonparametric one-way analysis of variance (ANOVA) was used with post-hoc intergroup comparisons by Tukey test where appropriate. For correlations between parameters, Pearson correlation tests were performed, reporting the correlation coefficients (r). Univariate analysis of overall survival was performed using the Kaplan-Meier method with the log-rank test. Multivariate regression analyses of survival data were based on Cox proportional hazards modeling. All statistical analyses were performed using SPSS version 21.0 (IBM Corporation, Armonk, New York), Sigmaplot (Systat Software, Inc., San Jose, CA), or the R statistical package (R version 2.13.0, The R Foundation for Statistical Computing, Vienna, Austria).

## Results

### Patient Demographics and Clinical Characteristics

The cohort consisted of 333 patients with metastatic prostate cancer treated with single-agent docetaxel. Ninety eight (29%) patients received low-dose weekly regimens, and 235 (71%) patients received non-weekly regimens. The majority (78%) of patients were either obese (*n* = 118) or overweight (*n* = 141), and 74 (22%) had normal BMI. There were no patients in the undernourished category. On the basis of the criteria of Martin et al. [24], only three patients were sarcopenic.

Table 1 summarizes baseline characteristics for all patients and by BMI category and type of chemotherapy regimen (weekly versus non-weekly). There were no statistically significant differences except for serum alkaline phosphatase, which was lower in patients treated with weekly regimens than other regimens (*P* = 0.009).

**Table 1:** Patient Characteristics.

BMI category		I: BMI<25 (N = 74)		II: BMI 25–30 (N = 141)		III: BMI>30 (N = 118)		Combined (N = 333)	*P*
Weekly Regimens		(N = 21)		(N = 43)		(N = 34)		(N = 98)	0.933
Other Regimens		(N = 53)		(N = 98)		(N = 84)		(N = 235)	
Black Race	Weekly	0.05 (1)	0.68	0.14 (6)	0.859	0.18 (6)	0.859	0.13 (13)	0.956
	Other	0.13 (7)		0.11 (11)		0.14 (12)		0.13 (30)	
Gleason Score (total)	Weekly	7/8/9	0.991	8/9/9	0.817	8/9/9	0.737	7/9/9	0.173
	Other	7/8/9		7/8/9		7/8/9		7/8/9	
P for BMI Category		I vs. II: 0.816		II vs. III: 0.985		I vs. III: 0.895			
Gleason Score ≤8	Weekly	0.43 (9)	0.823	0.53 (23)	0.594	0.53 (18)	0.303	0.51 (50)	0.387
	Other	0.49 (26)		0.47 (46)		0.40 (34)		0.45 (106)	
Alkaline Phosphatase	Weekly	81.0/115.0/213.0	0.952	61.0/ 88.0/132.5	0.289	70.0/ 80.5/105.8	0.727	63.5/ 88.5/134.8	0.009
	Other	80.0/129.0/222.0		76.8/100.5/228.8		73.5/ 99.5/145.2		76.0/104.0/197.0	
P for BMI Category		I vs. II: 0.208		II vs. III: 0.731		I vs. III: 0.06			
Alkaline Phosphatase >126	Weekly	0.48 (10)	1	0.28 (12)	0.477	0.15 (5)	0.069	0.28 (27)	0.081
	Other	0.51 (27)		0.36 (35)		0.33 (28)		0.38 (90)	
Age-unadjusted CCI	Weekly	6/7/9	0.998	6/6/8	1	6/7/8	1	6/6/8	0.915
	Other	6/7/8		6/6/8		6/7/8		6/7/8	
P for BMI Category		I vs. II: 0.818		II vs. III: 0.977		I vs. III: 0.729			
Age-unadjusted CCI ≤6	Weekly	0.52 (11)	0.827	0.44 (19)	0.818	0.53 (18)	0.741	0.49 (48)	0.47
	Other	0.58 (31)		0.48 (47)		0.58 (49)		0.54 (127)	

Age is given in years. Continuous and ordinal variables are presented as 1st quartile limit/median/3rd quartile limit. The count data are presented as proportions of the total in the group, with actual counts in parentheses. Proportions of count data were compared using Chi square test or Fisher’s exact test where appropriate. Continuous and ordinal data were analyzed using two-way analysis of variance (ANOVA).

**Fig 1:**
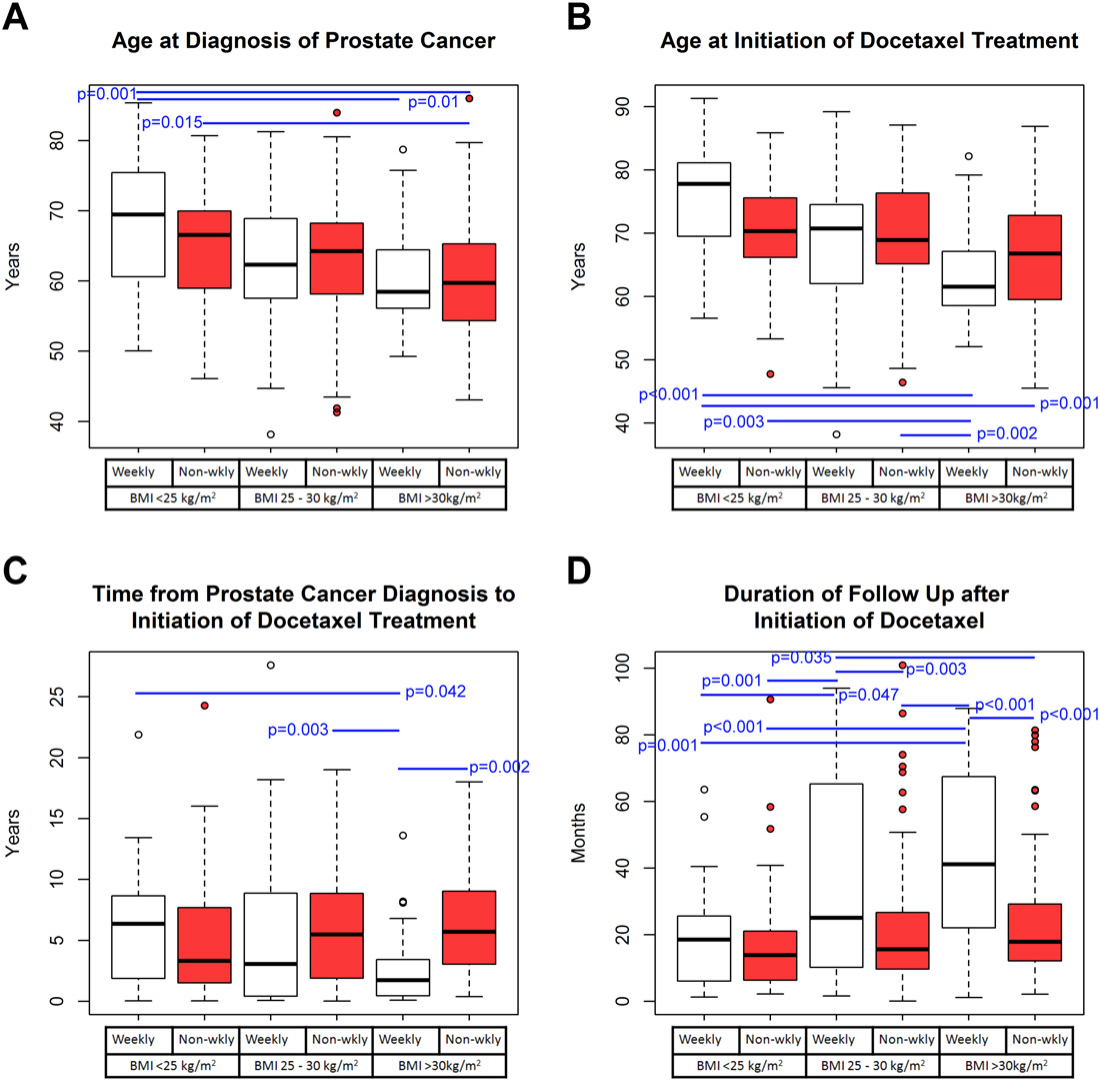
Time-related differences among groups of prostate cancer patients with different BMIs who received weekly docetaxel treatment or other docetaxel regimens. Box plots are shown for the following parameters: A) age at diagnosis of prostate cancer, B) age at initiation of docetaxel treatment, C) the number of years after diagnosis of prostate cancer when docetaxel treatment was initiated, and D) the number of months of follow-up starting from the initiation of docetaxel therapy. The black line inside each box represents the median, and outliers are represented by circles beyond the whiskers. The patient groups are as labeled beneath the horizontal axes. The groups that received weekly docetaxel regimens are shown in white, and those received other regimens are shown in red. The blue lines with *P* values labeled next to each indicate significant (*P* < 0.05) intergroup comparisons. The *P* values were based on non-parametric one-way ANOVA (Kruskal-Wallis) with post-hoc intergroup comparisons using the Tukey test.

At cancer diagnosis, obese patients on weekly regimens were significantly younger than patients with normal BMI on weekly regimens (*P* = 0.01) and obese patients on non-weekly regimens were significantly younger than patients with normal BMI (on weekly regimens: *P* = 0.001; on other regimens: *P* = 0.015) (Fig. 1A). At docetaxel initiation, obese patients on weekly regimens were significantly younger than patients with normal BMI (on weekly regimens: *P* < 0.001) and with normal BMI (*P* = 0.003) and overweight patients on non-weekly regimens (*P* = 0.002) (Fig. 1B). Time from diagnosis of prostate cancer to initiation of docetaxel treatment was significantly shorter in obese patients on weekly regimens than obese (*P* = 0.002), overweight patients (*P* = 0.003) on non-weekly regimens, and normal BMI patients on weekly regimens (*P* = 0.042) (Fig. 1C). The duration of follow-up after starting docetaxel was significantly longer in obese and overweight patients on weekly regimens than all the other groups (*P* < 0.05 for all the intergroup comparisons with the other groups; Fig. 1D). This increased duration of follow-up might be due to improved survival, and the possible association of weekly regimens with longer survival was further examined. Given that docetaxel is lipophilic, the body composition relating to fat mass was expected to influence the volume of distribution and docetaxel plasma concentrations. Therefore, we also investigated the associations of body composition parameters with clinical outcomes.

### Body Composition Parameters

**Fig 2:**
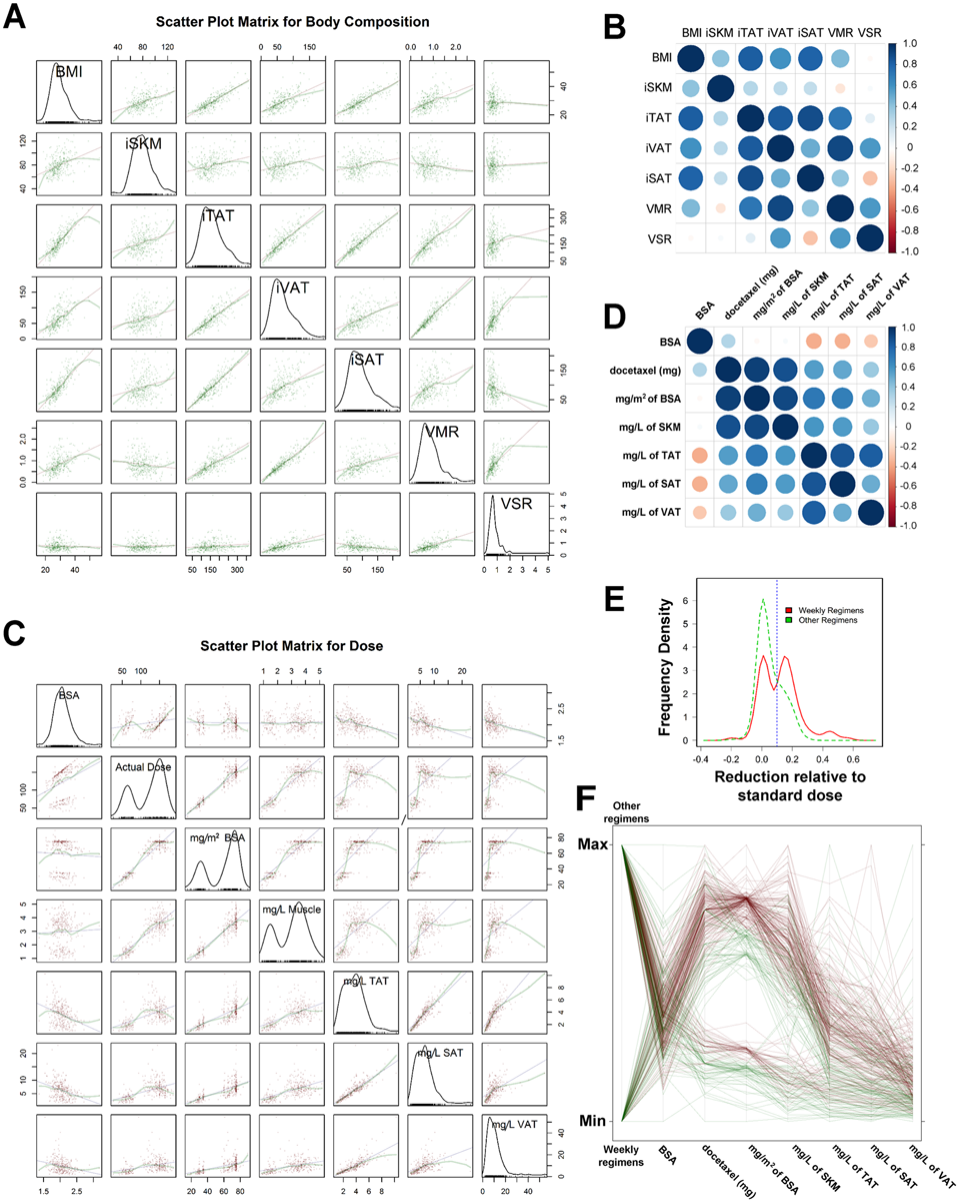
Correlations among body composition parameters and among docetaxel dosage calculations. A) A matrix of scatter plots for body composition parameters is shown. The frequency distribution of each body composition parameter is presented as labeled at the diagonal of the matrix. B) A correlation matrix of the body composition parameters is shown. The sizes of the dots indicate the magnitude of the correlation, and the dots are color coded for the correlation coefficient according to the scale on the right. The correlation coefficients are reported in the corresponding boxes reflected across the diagonal. C) A matrix of scatter plots for dosages calculated based on body composition parameters is shown. The frequency distribution of each parameter is presented as labeled at the diagonal of the matrix. D) A correlation matrix of the dosages is shown in a manner similar to that for B. E) The probability density function, estimated by kernel density estimation, of the proportion of the initial docetaxel dose relative to the reference dose for the weekly regimens (red solid line) and for the other regimens (green dashed line). The blue vertical dotted line represents a 10% reduction of the initial docetaxel dosage relative to the respective reference dosage for the groups as labeled. F) A parallel coordinates plot is drawn to demonstrate the relationships among covariates such as the type of docetaxel regimen, the dose of docetaxel in milligrams, and docetaxel dosages calculated based on body composition parameters. The patients with empirical reduction of the initial docetaxel dosage based on mg/m^2^ BSA by ≥10% are represented by green lines, and those otherwise are represented by brown lines.

The correlation was positive between BMI and adipose tissue (iTAT, iVAT, and iSAT) and skeletal muscle (iSKM), weak for VMR, and practically non-existent between BMI and VSR (Fig. 2A). The correlation matrix summarizes the correlation coefficients among these indices (Fig. 2B). Therefore, VMR and VSR add information about the body composition that cannot or can only poorly be predicted by BMI.

### Docetaxel Dosing and Body Composition parameters

The docetaxel doses were generally based on the recommended dosage per m^2^ of BSA. The frequency distributions of actual dose, the mg/m^2^ of BSA and mg/L of muscle were bimodal (Fig. 2C), since the majority of the patients received the FDA-approved dose of 75 mg/m^2^ given every 3 weeks, and a second group of patients received the weekly regimens with a peak near 35 mg/m^2^. However, 115 patients (34.5%) started docetaxel with empirical dosages that were <90% of the reference dosages of 35 mg/m^2^ and 75 mg/m^2^ for the weekly regimens and non-weekly regimens, respectively (i.e., empirical dosage reduction by >10%). The frequency distribution of docetaxel dose per calculated whole-body volumes (liter) of skeletal muscle (SKM) and total adipose tissue (TAT) is shown in Fig. 2C. The correlation matrix summarizes the correlation coefficients among these dosage calculations (Fig. 2D). Negative correlations of mg/L of TAT, SAT, or VAT with BSA were observed.

Body composition parameters of the patients in different docetaxel regimen categories are summarized in S1 Table. There were no significant differences between the weekly regimen group and the non-weekly regimen group. Comparing mg/m^2^ of docetaxel dose per BSA received by the patients against the standard dose for the respective regimens (i.e., 35 mg/m^2^ for the weekly regimen and 75 mg/m^2^ for non-weekly regimens), the mean relative reduction in dosage was significantly (*P* < 0.001) higher in the weekly regimen group (0.121) than the non-weekly regimen group (0.054). The probability density functions, estimated by kernel density estimation, for the two groups are shown in Fig. 2E; the group with weekly regimens was clearly bimodal with the relative dose reduction of 0.1 separating the two modes (peaks).

A parallel coordinates plot of weekly regimens (yes vs. no), BSA, mg quantity of docetaxel per dose, mg/m^2^ of BSA, mg/L of SKM, mg/L of TAT, mg/L of SAT, and mg/L of VAT is shown in Fig. 2F, It is obvious that the patients who received the weekly regimens had a significantly higher proportion (χ^2^ test, *P* < 0.001) of patients with >10% dose reduction (55/98) than those receiving non-weekly regimens (61/235).

### Body Composition Parameters and Survival

In univariate Kaplan-Meier analysis, differences in overall survival from diagnosis to death were evaluated for BMI categories; no statistically significant differences were observed. The median time from diagnosis of prostate cancer to initiation of docetaxel was 52.77 months (95% confidence interval [95% CI]: 41.53–64.01), and there were no significant differences among the BMI categories using the log-rank test.

**Fig 3:**
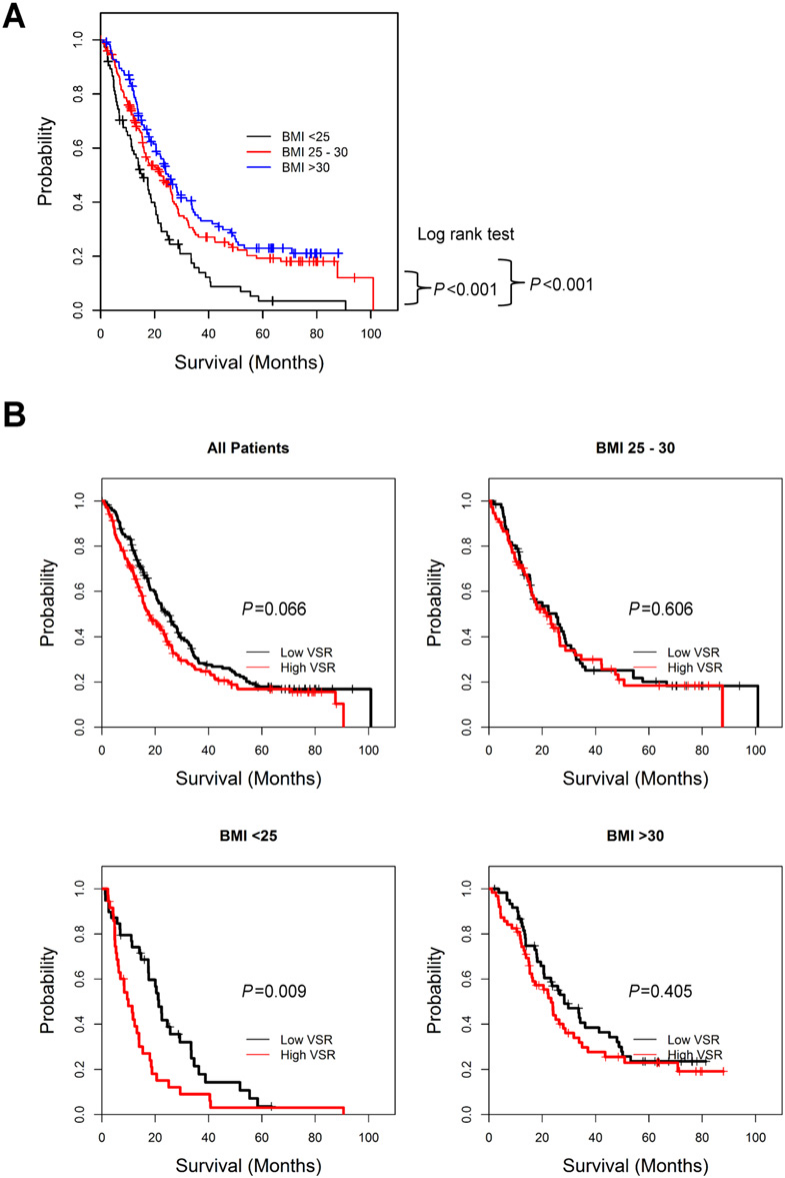
Overall survival of metastatic prostate cancer patients starting docetaxel treatment. A) Univariate Kaplan-Meier survival functions for overall survival starting from the initiation of docetaxel treatment to the event of death are shown for patients in different BMI categories as labeled. Each + represents a censored data point. B) Univariate Kaplan-Meier survival functions for overall survival are shown for all patients in the study cohort grouped by VSR (upper left). Stratified by BMI categories (<25, 25–30, and >30 kg/m^2^ for the lower left, upper right, and lower right subpanels, respectively), the survival functions for patients in each BMI category are shown for the different VSR groups as labeled. Each + represents a censored data point.

The time from the initiation of docetaxel to death for all patients was analyzed by the Kaplan-Meier method, and the median time was 21.1 months (95% CI: 17.80–24.40). Normal BMI patients had a shorter time from initiation of docetaxel treatment to death [median: 14.7 months; 95% confidence interval (95% CI): 10.32–19.08] than overweight (median: 22.27 months; 95% CI: 15.61–28.93) and obese patients (median: 25.87 months; 95% CI: 21.52–30.12) (log rank test; *P* < 0.001; Fig. 3A).

Using the median cut-off value for other body composition parameters, significant differences in survival were also observed for iSKM, iTAT, iVAT, and iSAT but not for VMR (S2 Table) as expected, as BMI correlated with iSKM, iTAT, iVAT, and iSAT but very poorly with VMR. Patients with high (above median) VSR had a near-significant (*P* = 0.066) difference of shorter survival time as compared with the low VSR group. When stratified by BMI categories, high VSR was associated with shorter survival (median: 9.9 months; 95% CI: 6.2–13.7) as compared to the low VSR group (median: 21.3 months; 95% CI: 18.1–24.5) in normal BMI patients (*P* = 0.009) but not in overweight (*P* = 0.606) or obese (*P* = 0.405) patients (Fig. 3B). No significant differences were found between high and low VMR stratified by BMI categories.

### Dosage and Regimens of Docetaxel and Survival

**Fig 4:**
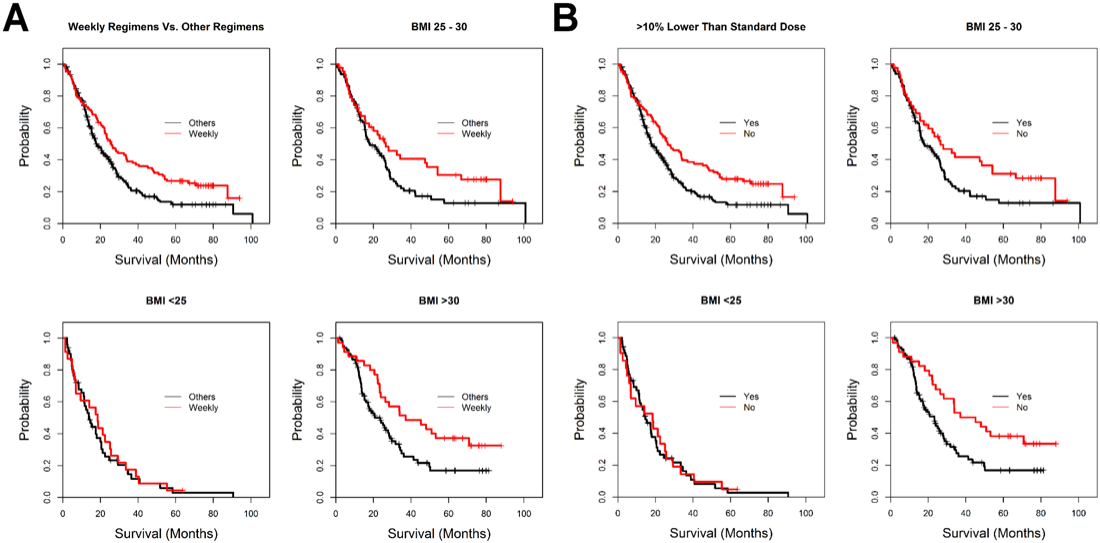
Overall survival of metastatic prostate cancer patients starting docetaxel treatment. A) Univariate Kaplan-Meier survival functions for overall survival starting from the initiation of docetaxel treatment to the event of death are shown for all the patients in the study cohort grouped by docetaxel regimen (upper left subpanel). Stratified by BMI categories (<25, 25–30, and >30 kg/m^2^ for the lower left, upper right, and lower right subpanels, respectively), the survival functions for patients in each BMI category are shown for the different docetaxel regimens as labeled. Each + represents a censored data point. B) Survival functions of overall survival are shown, in a manner similar to that shown in A, as grouped by reduction of the initial docetaxel dosage relative to the reference dose.

Patients who were treated with weekly regimens had longer survival (median: 26.6 months; 95% CI: 17.9–35.3) than the non-weekly regimen group (median: 17.8 months; 95% CI: 14.8–20.9; *P* = 0.002; Fig. 4A,). Stratification by BMI categories revealed that this association was present in overweight (*P* = 0.051) and obese patients (*P* = 0.011), but not in normal BMI patients (*P* = 0.842; Fig. 4A).

Patients (n = 115; 34.5%) who received docetaxel with empirical dosage reduction by >10% of the recommended dose, had significantly shorter survival (median: 18.2 months; 95% CI: 14.1–22.2) than those patients without such dosage reduction (median: 22.4 months; 95% CI: 17.9–26.8; *P* = 0.001; Fig. 4B). Again, stratification by BMI categories revealed that this association was present in overweight (*P* = 0.033) and obese patients (*P* = 0.001), but not in the normal BMI patients (*P* = 0.915; Fig. 4B).

### Multivariate Analysis of Factors Associated with Survival of Patients with Metastatic Prostate Cancer Receiving Docetaxel

A Cox proportional hazard regression model was constructed using known factors that predict prostate cancer survival, i.e., age, race, Gleason score, and survival for more than 5 years since the diagnosis of prostate cancer. Other factors included were: abnormal serum alkaline phosphatase, as an indicator of significant disease burden of bone metastasis; age-unadjusted CCI, to control for comorbidity; the type of regimen (weekly vs. non-weekly); whether the initial dose was reduced by >10% of a reference dosage, and any subsequent reduction of dosage intensity. The BMI, VMR, and VSR were the body composition parameters included in the model. We found old age (≥65 years), abnormal serum alkaline phosphatase, >10% reduction of initial dosage, and high VSR to be significant predictors of shorter survival time, while high VMR, obesity, and use of weekly regimens to be significant predictors of longer survival after docetaxel initiation (Table 2).

**Table 2:** Cox regression model for time between docetaxel initiation and death.

Model covariates	B	*P*	Exp(B)	95% CI for Exp(B)	
				Lower	Upper
≥5 Years after cancer diagnosis	-0.065	0.637	0.937	0.715	1.228
Age >65 years	0.373	0.013	1.452	1.082	1.950
African race	-.251	0.224	0.778	0.520	1.165
Gleason score > 8	0.105	0.444	1.111	0.849	1.455
Abnormal serum alkaline phosphatase	0.622	<0.001	1.862	1.415	2.451
VMR > Median	-0.343	0.040	0.710	0.512	0.984
VSR > Median	0.366	0.016	1.442	1.071	1.943
BMI Categories (overall)		0.111			
BMI = 25–30 kg/m^2^ vs. BMI <25 kg/m^2^	-0.256	0.124	0.774	0.558	1.073
BMI >30 kg/m^2^ vs. BMI <25 kg/m^2^	-0.389	0.040	0.678	0.468	0.982
Age-unadjusted CCI >6	0.035	0.797	1.036	0.793	1.354
Weekly regimen	-0.513	0.001	0.598	0.437	0.819
>10% reduction of initial dosage	0.501	0.001	1.651	1.244	2.192
Subsequent reduction in dosage intensity	-0.126	0.579	0.882	0.566	1.374

### Factors Associated with >10% Emperical Reduction of Initial Docetaxel Dosage and with Subsequent Reduction of Dosage Intensity

Using a logistic regression model, factors associated with >10% reduction of the initial docetaxel dose were presence of high comorbidity (age-unadjusted CCI ≥6; *P* = 0.01), use of weekly regimens (*P* < 0.001) and being overweight as compared to normal BMI (*P* = 0.02). (S3 Table). Age >65 years was a near significant (*P* = 0.061) predictor.

Potential factors for subsequent reduction of docetaxel dosage intensity were investigated by an expanded logistic regression model that also included the status of empirical reduction of dosage by >10% and the adverse reactions/toxicities of docetaxel. We found diarrhea to be a significant predictor (*P* = 0.014) (S3 Table), while mucositis and hand-foot syndrome were near-significant predictors (*P* = 0.077 and *P* = 0.075, respectively). Being obese was a significant negative predictor (*P* = 0.016, compared with normal BMI). Being overweight was a near-significant negative predictor (*P* = 0.098, compared with normal BMI).

## Discussion

Obesity is a worsening worldwide problem costing 2 trillion US dollars annually [26]. While evidence is accumulating about the association of obesity with prostate cancer aggressiveness and poor clinical outcomes [27], little is known about any influence of body composition on clinical benefits of specific therapies for prostate cancer. Our retrospective study examined the association between BMI and body composition characteristics in terms of muscle mass, adipose tissue and visceral obesity, with clinical outcomes (toxicity and survival) in a cohort of patients with metastatic prostate cancer who received docetaxel treatment. We found that the overweight and obese patients were diagnosed at a younger age and developed metastatic disease that was treated with docetaxel earlier than normal BMI patients, but they died at about the same time after diagnosis as the normal BMI patients. In overweight and obese patients, treatment with weekly regimens was associated with longer survival from initiation of treatment to death than non-weekly regimens.

The improved survival benefit of docetaxel, especially the weekly regimens, in overweight and obese patients may partly be explained by docetaxel pharmacokinetics, which are similarin the weekly and triweekly regimens [12]. While this manuscript was under review, Cushen et al. reported in a poster at the 2014 European Society of Medical Oncology Meeting that high BMI was associated with longer survival in patients with castration resistant prostate cancer taking docetaxel [28], and corroborated our finding. Weekly regimens may possess antiangiogenic properties relative to triweekly regimens because the plasma concentration of docetaxel throughout the weekly treatment is maintained above 1 nM [12], which is approximately the concentration that inhibits angiogenesis by 50% [29, 30]. Docetaxel is highly lipophilic, and drugs with high affinity for adipose tissue have increased volumes of distribution (Vd) in obese patients [31]. While the clearance of docetaxel might not be changed in obesity, the steady state Vd and the elimination half-life (t½) of the terminal phase are increased in the obese compared with non-obese patients [15, 32], low peak plasma concentrations and prolonged presence of low levels may be the mechanistic basis for a better metronomic therapeutic effect of weekly docetaxel in obese than non-obese patients.

In 2010, a survey revealed a 46% prevalence of empirical dose adjustment of chemotherapy in obese patients [33]; empirical dose reduction was most likely to occur in patients with increased age, severe obesity, and poor performance status. Although ASCO subsequently published guidelines recommending dosing of chemotherapy based on body surface area using actual weight in obese adults [19], how the practice of empirical dose adjustment has changed is unclear. We found that at our institution about one third (34.5%) of patients had significant empirical dose reductions of 10% or higher at docetaxel initiation. In our study cohort, high comorbidity, overweight or obese status, and weekly docetaxel regimens were the factors associated with empirical reduction by >10%, while old age was a near significant factor. Additional potential reasons for empirical reduction included the practice of BSA capping, and different dosage regimens in published papers that differ by >10%. Seminal papers have used different docetaxel dosages. Tannock et al. used docetaxel 75 mg/m^2^ every 3 weeks [5], but Petrylak et al. used docetaxel 60 mg/m^2^ every 3 weeks [6], which is 20% lower than used by Tannock et al. While we observed no difference in survival after initiation of docetaxel in normal BMI patients with or without >10% empirical reduction of initial docetaxel dosage, the possible difference in efficacy between the reference dosage and reduced dosages will need to be further investigated in overweight and obese men with metastatic prostate cancer. We found negative correlations of dosage mg/L of TAT, SAT, or VAT with mg/m^2^ BSA suggested that our clinicians tended to prescribe a lower dosage on a mg/m^2^ BSA basis for obese patients (Fig. 2C and 2D) or to cap the BSA when calculating the initial dose of docetaxel as practiced by some clinicians [34]. These reductions in obese and overweight subjects but not in normal BMI was associated with shortened survival. One plausible explanation is that obesity, by increasing the half-life of the terminal phase, increases the trough level of docetaxel to approach a metronomic therapeutic effect, which may be compromised by >10% dosage reduction.

Subsequent reduction of docetaxel dosage intensity may be viewed as an indicator of unacceptable toxicity or adverse reaction. Our logistic regression showed that it was associated with diarrhea and probably also mucositis and hand-foot syndrome. Being obese was associated with fewer occurrences of subsequent reduction of dosage intensity than normal BMI. Possible explanations were that obese patients could tolerate docetaxel better than normal BMI patients or that obese patients were treated with initial docetaxel doses that were reduced empirically mg/m^2^ and therefore had fewer side effects and toxicity that prompted subsequent dosage reduction. Therefore, these results for docetaxel in male patients are consistent with the findings by Lyman and Sparreboom that chemotherapy doses based on BSA calculated from actual body weights did not cause more toxicity in obese patients than in normal BMI patients, and empirical reduction of dosage for obese patients to reduce toxicity was not warranted [35].

Gurney and Shaw eloquently discussed the dilemma of dose calculation for obese patients [36]. Obesity and body fat distribution are only two factors out of many that lead to inter-patient variability in pharmacokinetics [34]. The important issue is whether these differences related to body composition actually translate into differences in treatment efficacy and clinical outcome. Our multivariate Cox regression model found that >10% reduction of the initial docetaxel dosage was associated with shortened time from the initiation of docetaxel to death. This suggests that empirical docetaxel dosage reduction for obese men with metastatic prostate cancer might cause harm. The toxicity-adjusted dosing approach may be prudent.

There is increasing evidence of visceral obesity and poor outcomes after cancer diagnosis [37–40]. Although BMI correlated with iSKM, iTAT, iVAT, and iSAT, it predicts the distribution of body mass between the adipose and muscle compartments poorly and does not predict the visceral and subcutaneous fat distribution at all. Therefore, VMR and VSR are two body composition parameters that will provide additional information not conveyed by BMI. Given the same BMI, a patient with a muscular build may have different pharmacokinetic characteristics from one with central obesity from hypogonadism or hypercortisolism. However, data is lacking to relate body composition parameters such as VSR and VMR to pharmacokinetic parameters. Our stratified Kaplan-Meier analysis showed that VSR above median was associated with decreased survival from initiation of docetaxel treatment in normal BMI patients (Fig. 2B). Whether this is due to subtle changes in docetaxel pharmacokinetics caused by a different distribution of visceral and subcutaneous fat or changes in inflammation and hyperinsulinemia (parts of the metabolic syndrome) associated with high VSR remains to be further investigated.

In summary, our retrospective analysis of body composition of men with metastatic prostate cancer treated with docetaxel found the presence of obesity and the administration of weekly docetaxel regimens to be associated with longer survival, whereas dose reductions in docetaxel at therapy initiation and the presence of visceral obesity were associated with poorer survival. Obese patients may benefit more from weekly docetaxel regimens using the reference dosage of 35 mg/m^2^ than non-obese patients. While it is not practical to change the composition of the body before docetaxel treatment, one interesting hypothesis for future research is whether changing the dosage and dosing schedule to keep the trough level of docetaxel above 1 nM [12] can improve the efficacy in non-obese patients compared with the weekly docetaxel regimen. Future research should obtain data to relate body composition parameters to pharmacokinetics in order to provide evidence-based guidance to custom-fit docetaxel dosage regimens for patients with different body sizes and composition.

## Supporting Information

S1 Methods Supplemental Methods. (DOCX)

S1 Table Body composition parameters of metastatic prostate cancer patients starting docetaxel chemotherapy (DOCX)

S2 Table Univariate Kaplan-Meier analyses of the association of body composition parameters of patients with metastatic prostate cancer with survival duration between docetaxel initiation and death (DOCX)

S3 Table Logistic regression models to predict empirical reduction of initial docetaxel dosage by >10% and subsequent reduction of dosage intensity (DOCX)
