# Supplementary material for: Association of Body Composition with Outcome of Docetaxel Chemotherapy in Metastatic Prostate Cancer: A Retrospective Review
Source: PLoS One. 2015 Mar 30;10(3):e0122047. doi: 10.1371/journal.pone.0122047 (PMC4379069; doi:10.1371/journal.pone.0122047)
Supplement: S1 Methods — (DOCX) [file pone.0122047.s001.docx]

**Supplemental Methods:**

*Body Composition of Patients with Metastatic Prostate Cancer*

Reliability of measurements of these parameters was evaluated by absolute percent differences between measurements of different image slices from the same patient and by absolute percent differences between measurements for the same patient by different reviewers.

The within-reviewer variabilities were 4.21% ± 4.38% (mean ± standard deviation) for muscle area, 2.19% ± 3.13% for total adipose tissue area, and 3.56% ±6.25% for visceral adipose tissue area. Inter-reviewer variabilities were 3.36% ± 3.20% for muscle area, 1.69% ± 1.97% for total adipose tissue area, and 2.84% ±5.61% for visceral adipose tissue area.
