# Supplementary material for: Association of Body Composition with Outcome of Docetaxel Chemotherapy in Metastatic Prostate Cancer: A Retrospective Review
Source: PLoS One. 2015 Mar 30;10(3):e0122047. doi: 10.1371/journal.pone.0122047 (PMC4379069; doi:10.1371/journal.pone.0122047)
Supplement: S1 Table — (DOCX) [file pone.0122047.s002.docx]

**Supplemental Table 1.** Body composition parameters of metastatic prostate cancer patients starting docetaxel chemotherapy

|  | Weekly regimens | Other regimens |  | All patients combined |
| --- | --- | --- | --- | --- |
|  | (*N* = 98) | (*N* = 235) | *P* | (*N* = 333) |
| BSA (m^2^) | 1.89/2.06/2.20 | 1.91/2.05/2.19 | 1.000 | 1.90/2.05/2.19 |
| BMI (kg/m^2^) | 25.43/27.96/32.73 | 25.34/28.33/32.22 | 0.709 | 25.35/28.31/32.31 |
| iSKM (cm^2^/m^2^) | 68.07/80.11/93.33 | 69.01/77.33/85.39 | 0.103 | 68.78/77.94/87.14 |
| iTAT (cm^2^/m^2^) | 111.28/150.20/182.34 | 119.09/154.60/197.23 | 0.236 | 115.47/153.07/190.38 |
| iSAT (cm^2^/m^2^) | 65.51/84.28/109.80 | 67.17/87.65/115.61 | 0.357 | 66.28/86.82/111.83 |
| iVAT (cm^2^/m^2^) | 42.76/55.93/77.24 | 41.80/61.56/85.26 | 0.264 | 41.81/60.12/83.92 |
| VMR | 0.51/0.72/0.98 | 0.58/0.79/1.06 | 0.091 | 0.57/0.77/1.06 |
| VSR | 0.51/0.66/0.88 | 0.52/0.67/0.86 | 0.726 | 0.52/0.67/0.88 |

The values are presented as 1st quartile limit/median/3rd quartile limit. The *P* values for comparing weekly regimens with other regimens were obtained using Mann-Whitney rank sum test.
