# Supplementary material for: Association of Body Composition with Outcome of Docetaxel Chemotherapy in Metastatic Prostate Cancer: A Retrospective Review
Source: PLoS One. 2015 Mar 30;10(3):e0122047. doi: 10.1371/journal.pone.0122047 (PMC4379069; doi:10.1371/journal.pone.0122047)
Supplement: S2 Table — (DOCX) [file pone.0122047.s003.docx]

**Supplemental Table 2.** Univariate Kaplan-Meier analyses of the association of body composition parameters of patients with metastatic prostate cancer with survival duration between docetaxel initiation and death

| Parameter | Group | Median survival (months) | 95% Confidence interval | Log rank test |
| --- | --- | --- | --- | --- |
| iSKM | High | 23.37 | 18.98–27.76 | *P* = 0.005 |
|  | Low | 17.97 | 14.26–21.68 |  |
| iTAT | High | 23.37 | 18.55–28.19 | *P* = 0.010 |
|  | Low | 18.77 | 15.45–22.09 |  |
| iVAT | High | 23.37 | 18.55–28.19 | *P* = 0.010 |
|  | Low | 18.77 | 15.45–22.09 |  |
| iSAT | High | 24.03 | 19.23–28.83 | *P* = 0.029 |
|  | Low | 17.70 | 14.22–21.18 |  |
| VMR | High | 23.20 | 18.56–27.84 | *P* = 0.245 |
|  | Low | 20.53 | 16.97–24.09 |  |
| VSR | High | 17.30 | 13.06–21.54 | *P* = 0.066 |
|  | Low | 24.60 | 20.37–28.83 |  |
