# Supplementary material for: Association of Body Composition with Outcome of Docetaxel Chemotherapy in Metastatic Prostate Cancer: A Retrospective Review
Source: PLoS One. 2015 Mar 30;10(3):e0122047. doi: 10.1371/journal.pone.0122047 (PMC4379069; doi:10.1371/journal.pone.0122047)
Supplement: S3 Table — (DOCX) [file pone.0122047.s004.docx]

**Supplemental Table 3.** Logistic regression models to predict empirical reduction of initial docetaxel dosage by >10% and subsequent reduction of dosage intensity

|  | **Empirical reduction of initial dosage by >10%** | | | **Subsequent reduction of dosage intensity** | | |
| --- | --- | --- | --- | --- | --- | --- |
| Covariates | B | *P* | Exp(B) | B | *P* | Exp(B) |
| Black race | 0.378 | 0.297 | 1.460 | -0.019 | 0.976 | 0.981 |
| Age >65 years | 0.553 | 0.061 | 1.739 | -0.637 | 0.200 | 0.529 |
| Abnormal serum alkaline phosphatase | 0.055 | 0.841 | 1.056 | -0.669 | 0.184 | 0.512 |
| VMR > Median | 0.122 | 0.702 | 1.130 | 0.268 | 0.626 | 1.308 |
| VSR > Median | -0.147 | 0.620 | 0.863 | 0.086 | 0.867 | 1.089 |
| BMI categories (overall) |  | 0.007 |  |  | 0.052 |  |
| BMI = 25–30 kg/m^2^ vs. BMI <25 kg/m^2^ | -0.803 | 0.020 | 0.448 | -0.927 | 0.098 | 0.396 |
| BMI >30 kg/m^2^ vs. BMI <25 kg/m^2^ | 0.063 | 0.867 | 1.065 | -1.595 | 0.016 | 0.203 |
| Gleason score >8 | -0.313 | 0.219 | 0.731 | -0.389 | 0.392 | 0.677 |
| Age-unadjusted CCI >6 | 0.679 | 0.010 | 1.972 | 0.468 | 0.304 | 1.596 |
| Weekly regimens | 1.567 | <0.001 | 4.790 | 0.465 | 0.331 | 1.591 |
| Empirical reduction of initial dosage by >10% of reference dosage |  |  |  | -0.304 | 0.547 | 0.738 |
| Mucositis |  |  |  | 1.337 | 0.077 | 3.808 |
| Diarrhea |  |  |  | 1.147 | 0.014 | 3.147 |
| Nausea/vomiting |  |  |  | -0.215 | 0.762 | 0.807 |
| Allergic reactions |  |  |  | 0.592 | 0.396 | 1.808 |
| Hand-foot syndrome |  |  |  | 1.554 | 0.075 | 4.732 |
| Neuropathy |  |  |  | 0.229 | 0.655 | 1.258 |
| Neutropenia |  |  |  | -0.335 | 0.687 | 0.715 |
| Constant | -1.508 | 0.001 | 0.221 | -1.735 | 0.013 | 0.176 |
